# Multiple Sclerosis and Cancer: The Ying-Yang Effect of Disease Modifying Therapies

**DOI:** 10.3389/fimmu.2019.02954

**Published:** 2020-01-10

**Authors:** Esther Melamed, Michael William Lee

**Affiliations:** ^1^Department of Neurology, Dell Medical School, Austin, TX, United States; ^2^Department of Oncology, Department of Medical Education, Dell Medical School, Austin, TX, United States

**Keywords:** multiple sclerosis, disease modifying therapy, cancer, treatment of autoimmune disease, multiple sclerosis drug mechanism, cancer treatment, multiple sclerosis treatment

## Abstract

Over the past two decades, the field of multiple sclerosis (MS) has been transformed by the rapidly expanding arsenal of new disease modifying therapies (DMTs). Current DMTs for MS aim to modulate innate and adaptive immune responses toward a less inflammatory phenotype. Since the immune system is also critical for identifying and eliminating malignant cells, immunosuppression from DMTs may predictably increase the risk of cancer development in MS patients. Compared with healthy controls, patients with autoimmune conditions, such as MS, may already have a higher risk of developing certain malignancies and this risk may further be magnified by DMT treatments. For those patients who develop both MS and cancer, these comorbid presentations create a challenge for clinicians on how to therapeutically address management of cancer in the context of MS autoimmunity. As there are currently no accepted guidelines for managing MS patients with prior history of or newly developed malignancy, we undertook this review to evaluate the molecular mechanisms of current DMTs and their potential for instigating and treating cancer in patients living with MS.

## Introduction

**Figure 1:**
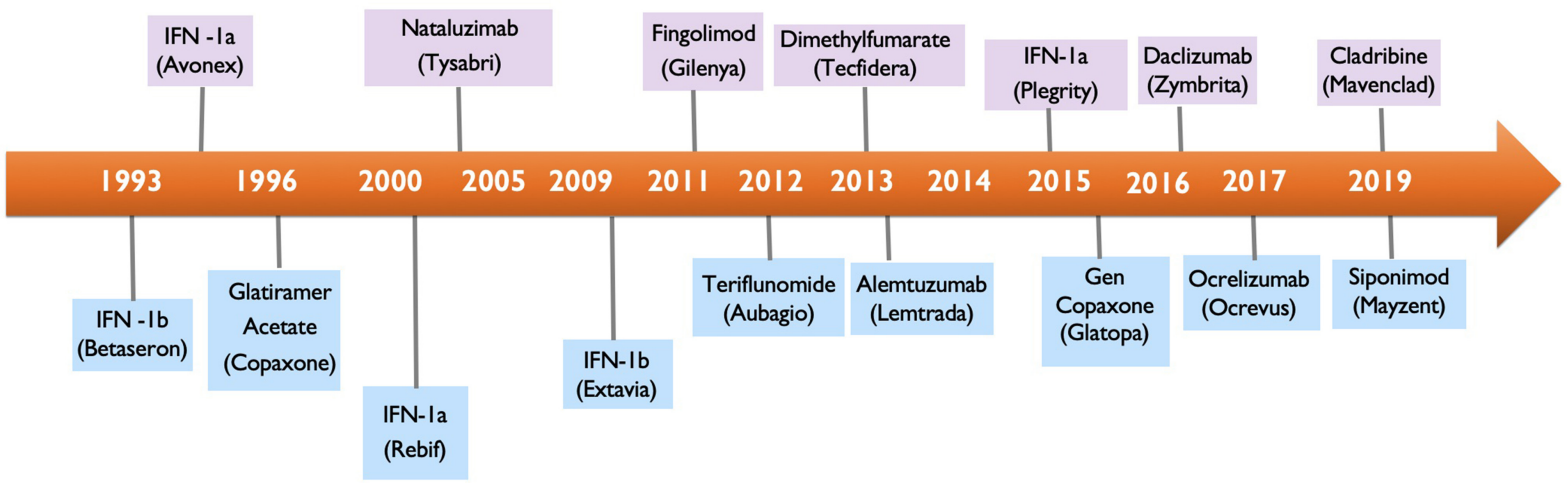
Multiple sclerosis drug approval timeline.

Multiple Sclerosis (MS) is a chronic autoimmune demyelinating disease of the central nervous system (CNS) and the leading non-traumatic cause of neurological disability in young adults. At diagnosis, most patients are started on a DMT, an immunomodulating or immunosuppressive therapy, that will likely be continued for life. While the DMT therapies have offered substantial benefit for MS patients, they have also introduced the potential for causing cancer as an adverse effect. With 17 DMTs available for the treatment of MS (Figure 1), clinicians are faced with the critical decision of how to screen MS patients for potential risk of malignancy with currently available DMTs and how to manage DMTs in MS patients who either had prior history of cancer or newly develop cancer while on a DMT.

The immune system plays an important role in both MS and cancer. It is possible that activation of the immune system in MS results in protective effects against cancer by increasing immunosurveillance, while chronic inflammation and use of certain immunosuppressive therapies could result in loss of immune protection against cancer or activation of the immune system to become pro-tumoragenic (1). At the same time, many of the available MS DMTs had been used for years as cancer treatments prior to being re-purposed for MS, such as rituximab, cladribine, and methotrexate, while other DMTs are actively being evaluated for their anti-tumor potential, such as dimethyl fumerate, fingolimod, and teroflunomide. This interesting dichotomy between the potential for cancer induction and cancer inhibition suggests that immunomodulatory and immunosuppressive MS medications may be acting in a context dependent manner in susceptible individuals.

The data on cancer prevalence and incidence in MS patients has been conflicting. Prior to the advent of oral and intravenous (IV) DMTs, studies suggested that the risk of cancer was equal (2, 3) or lower (4–6) in untreated MS patients or in MS patients treated with interferons and Copaxone, while other studies suggested a higher risk of certain cancers. Incidence of breast cancers has been found to be increased (2, 3, 7, 8), decreased (5), or the same (9, 10) in MS patients. Gastrointestinal cancers have been noted to be higher or lower in MS populations (1, 3). Urogenital (3, 11), central nervous system (8, 12), and skin cancers (1, 13) appear to be higher in the MS population. Several studies have found that while cancer risk in MS patients may not differ from the general population, the risk appears to be lower in male patients compared to female patients, and increases with age (7, 14, 15). Overall, there is general agreement that immunosuppressant therapies, such as azathioprine, methotrexate, cyclophosphamide, and mitoxantrone have led to increased cancer risks in MS patients and this risk is related to patient's family history of cancer, duration of treatment and cumulative dose (16, 17). More recent large cohort studies, have incorporated oral and IV therapies along with other cancer risk factors, such as alcohol and smoking, demonstrating no increase in cancer risk in MS population (7, 18, 19). Varying study outcomes have been attributed to differences in study design, methodology, sex of participants, accountability for environmental cancer risk factors, and participant geographical location. In addition, lack of long-term safety registries overseen by a central body, have led to bias in reporting and assessment of how cancer risk compares to the risk for the general population across different countries.

Our goal in this review is to concisely summarize the molecular mechanism of available DMTs and their mechanistic relationship with potential for causing and treating cancer. We hope that this review will be of interest to clinicians caring for patients with MS, basic science immunologists, oncologists, and cancer biologists.

## First Approved Disease Modifying Therapies in MS

### Interferon-β Therapies: Betaseron, Avonex, Rebif, Plegridy

Interferon-β (IFN-β) therapies revolutionized management of MS as the first MS specific DMTs, following their initial approval in 1990's. Interferons differ in the frequency and mode of administration, for example betaseron, rebif, and plegridy are subcutaneous, while avonex is intramuscular. The pegylated IFN-β-1a, plegridy, has a long half-life and is administered once every 2 weeks. IFN-β-1b is made and purified from *Escherichia coli*, and has amino acid differences from the body's own IFN-β, while IFN-β-1a is manufactured in the Chinese hamster ovary cell line and has the same protein sequence as the native IFN-β.

Interferons are cytokines that are naturally produced in the body in response to immune stimulation, viral infections, and other chemical stimulation (20). Isaacs, Lindenmann, Paucker, and others, defined interferons as antiviral agents with potential for antigrowth and anti-inflammatory activity. IFNs are now classified as Type I (IFN-α, β, and ω), Type II (IFN-γ) or Type III (IFN-λ). Although both IFN-α and IFN-β have been studied in MS, IFN-β therapies have been shown to be superior in the management of MS, likely due to their higher immunoregulatory actions and less severe adverse effects profile (21–23). It is noteworthy though that a preserved *in vivo* response to IFN-α has been observed in MS patients with neutralizing antibodies against interferon-beta and that IFN-α2a reduces MRI disease activity in relapsing-remitting multiple sclerosis (RRMS) (22). IFNα has been shown to be an important anti-viral therapy in the treatment of hepatitis B and C, HIV, herpes zoster, as well as in the management of different malignancies, including melanoma, chronic myelogenous leukemia (CML), B cell leukemia (BLL), follicular lymphoma, non-Hodgkin's lymphoma, mycosis fungoides, multiple myeloma, AIDS-related Kaposi's sarcoma, carcinoid, and also bladder, renal, epithelial ovarian, and skin cancer (24). IFN-β-1a has also been used in the treatment of adrenocortical and carcinoid cancers (25, 26).

Mechanistically, type I interferons signal through interferon alpha/beta receptor-1 (IFNAR1) and interferon alpha/beta receptor-2 (IFNAR2), leading to activation of tyrosine kinase 2 (Tyk2) and janus kinase-1 (JAK1), signal transducer and activated transcription-1 (STAT1) and signal transducer and activated transcription-1 (STAT2) phosphorylation cascades, and ultimately activation of hundreds of genes important in IFN mediated immune and antiproliferative functions (27). In MS, IFN-β is thought to down-regulate major histocompatibility complex II (MHC II) expression and decrease lymphocyte activation (28). IFN-β mediated increases in apoptotic markers, Annexin-V and caspase-3, leads to specific B memory cell depletion. Additional mechanisms for IFN-β include downregulation of adhesion molecules such as very late adhesion-4 (VLA-4), it's ligand vascular cell adhesion moleculae-1 (VCAM-1), and matrix metalloproteinase (MMP), resulting in lower transmigration of lymphocytes across the blood–brain barrier (23). Activation of STAT1/STAT2 also contributes to secretion of anti- inflammatory cytokines, e.g. Interleukin 10 (IL-10), which can shift the immune profile toward anti-inflammatory T helper 2 (Th2) cells (29).

Both immune cells and tumor cells can produce interferons in a complex interplay. Type I interferons, such as IFN-α and IFN-β, produced by plasmacytoid dendritic cells can lead to multiple, diverse, downstream actions (24). These include upregulation of MHC I on APCs and expression of tumor cell antigens (30, 31), differentiation of CD8+ T cells into cytolytic effector cells (32), downregulation of T regulatory cells (33), reduction in IL-12p40 (34), and upregulation of IL15 together with further lymphocyte expansion (30). Type 1 IFN-orchestrated actions contribute toward inhibition of tumor cell differentiation, proliferation, migration and an increase in tumor cell death. IFN-α and -β can inhibit tumor cell growth in different malignancies in specific ways. For example, in neuroblastoma, IFN-β can induce apoptosis via downregulation of phosphatidylinositol 3-kinase/protein kinase B signaling (35). In melanoma and breast cancer, IFN-β induces cell death via the extrinsic TNF-related-apoptosis-inducing-ligand (TRAIL)-dependent pathway (36). In cervical cancer, Type I interferons signal via the extrinsic cellular FLICE (FADD-like IL-1β-converting enzyme)-inhibitory protein (cFLIP) and caspase-8 ligands (37). Interestingly, tumor cells, by means of somatic copy number alterations (SCNA), can “turn off” IFN-α and IFN-β production by homozygously deleting their respective genes (38). These mechanisms could potentially allow cancer cells to evade the immune system and metastasize.

There were no cancers associated with IFN-β in MS clinical trials. However, since the initial Federal Drug Administration (FDA) approval of IFN-β, there has been a trend for breast cancer noted in a study of the British Columbia MS database, evaluating a cohort of 5146 relapsing-onset MS patients and 48,705 person-years of follow-up, that did not reach statistical significance (39).

#### Glatiramer Acetate (Copaxone)

Glatiramer acetate (GA), was approved in 1996 in the US and in 2001 in Europe for RRMS and became the second non-interferon DMT to be approved for MS. It is an amino acid polymer, originally developed as an agent to mimic myelin basic protein in an effort to induce autoimmune encephalomyelitis (EAE) in an MS mouse model (40). The result of administrating GA to mice was a paradoxical improvement in EAE, and these studies paved the way toward open-label MS trials in patients (41). GA is administered subcutaneously.

The mechanism of action of GA is not fully understood, and likely involves activation of both the innate and adaptive immune systems, upregulation of anti-inflammatory M2 monocytes, Th2 cells and T regulatory cells (Tregs) (42). Studies using radiolabeled GA demonstrate that the gastrointestinal tract and thyroid gland contain the highest GA levels, with lowest levels in the CNS. GA and its metabolites are hydrophilic, which might prevent its crossing the blood–brain barrier, pointing toward largely peripheral actions of GA in MS (43).

Despite relatively lower efficacy in disease modulation, GA is considered to have a strong safety profile and no cancers were reported in clinical trials. In the post-marketing era surveillance, one study found an increased relative risk for breast cancer for females, though this was not statistically significant (9). Other studies have not found a significant association between GA and breast cancer (16, 39). Several skin cancers have been reported in patients on GA, including one case study of primary cutaneous anaplastic large- cell lymphoma and one case study of melanoma (44, 45).

## Lymphocyte Trafficking Interruption: Natalizumab and Sphingosine-1-Phosphate Receptor Agonists

### Natalizumab (Tysabri)

Natalizumab is the first monoclonal antibody for management of MS, approved in 2004, and remains one of the most potent DMTs in MS. By selectively blocking lymphocyte α4β1 integrin, natalizumab effectively prevents lymphocyte transmigration across the blood brain barrier to the CNS (46).

Natalizumab would appear to be a promising anti-cancer drug due to its ability to block cell adhesion. For example, α4β1 integrin is necessary for melanoma metastasis into lymph nodes and integrins are important for tumor angiogenesis (47). Although natalizumab has been considered as a treatment for multiple myeloma (48) and certain stages of melanoma (49), it has not overall proven to be a successful candidate in cancer therapeutics. Part of the reason is thought to relate to differences in leukocyte and cancer cell extravasation into tissues. Whereas, leukocytes heavily rely on integrins for migration into inflamed tissues, cancer cells have evolved complex and varied approaches to interact with their microenvironment for metastasis. Cancer cells either do not use integrins due to transition from mesenchymal to ameboid forms, can express multiple adhesive receptors, or can switch from the use of one integrin to another for adhesion and metastasis (50).

Several types of cancers have been reported with natalizumab use, including melanoma, breast cancer and diffuse large B cell lymphoma (Table 1) (52). The mechanism to explain susceptibility to natalizumab-associated cancers likely has to do with decreased T cell migration to tumor sites due to blocking of α4 integrin which interferes with antigen-specific T cells activation (78). Interestingly, α4β1 integrin may also play a role in reducing the invasive potential of melanoma cell lines (79).

**Table 1:** Summary of disease modifying therapies, their mechanisms of action, incidence of cancer in clinical trials, and studies of DMTs in different cancer types.

**Agent**	**Mechanism of action**	**Cancer incidence in MS patients in clinical trials**	**References**	**Assessed in cancer types**	**References**
		**Type(s)**		**Type(s)**	
Interferons	Activates IFN receptor linked JAK/STAT pathways leading to alteration of transcription of immune and antiproliferative genes; reduces migration of lymphocytes across the blood brain barrier	None		Breast, glioma, nasopharyngeal carcinoma, neuroblastoma, adrenocortical, pancreatic, and carcinoid cancers	(24, 51)
Glatiramer acetate	Amino acid polymer; activation of innate; and adaptive immune system; shift toward more protective Th2 immunity	None			
Natalizumab	Monoclonal antibody against a4-integrin; binds and blocks interaction of a4-integrin with ligands, preventing lymphocyte transmigration across the blood brain barrier	**AFFIRM:** RMS (*N* = 627): breast cancer (*N* = 1), cervical cancer (*N* = 1), metastatic melanoma (*N* = 1)	(52)	Multiple myeloma and melanoma	(48, 49)
Fingolimod	sphingosine 1 phosphate receptor (S1PR) modulator	**FREEDOMS II:** RRMS (*N* = 728):basal cell carcinoma (*N* = 16), squamous cell carcinoma (*N* = 4), uterine leiomyoma (*N* = 1), thyroid cancer (*N* = 1) **TRANSFORMS:** RRMS (*N* = 857): basal cell carcinoma (*N* = 5), melanoma *in situ* (*N* = 3), breast cancer (*N* = 4) RMS (*N* = 188): basal cell carcinoma (*N* = 1), squamous cell carcinoma (*N* = 1), malignant melanoma (*N* = 1) **INFORMS:** PPMS (*N* = 336): basal cell carcinoma (*N* = 14), squamous cell carcinoma (*N* = 6), malignant melanoma (*N* = 1), breast cancer (*N* = 1), non-Hodgkin's lymphoma (*N* = 1), malignant lung cancer (*N* = 1), ovarian cancer (*N* = 1), prostate cancer (*N* = 1)	(53) (54, 55) (56)	Breast cancer, lung, gastric tumors, and metastatic melanoma	(57, 58)
Siponimod	sphingosine 1 phosphate receptor (S1PR) modulator	**EXPAND:** SPMS (*N* = 1,099): unspecified skin cancer (*N* = 14 cases)	(59)		
Teriflunomide	Inhibitor of mitochondrial *de novo* pyrimidine synthesis enzyme dihydroorooate dehydrogenase; reduces lymphocytes in circulation	RRMS (*N* = 725): cervical carcinoma *in situ* (*N* = 1) RRMS (*N* = 111): uterine leiomyosarcoma (*N* = 1)	(60, 61)	Breast cancer	(62)
Cladribine	Nucleoside analog, inhibits DNA synthesis and DNA chain termination; cytotoxic particularly for lymphocytes and monocytes	**CLARITY:** RMS (*N* = 889): benign uterine leiomyosarcoma benign uterine leiomyosarcoma (*N* = 5), cervical carcinoma *in situ* (*N* = 1), melanoma (*N* = 1), ovarian carcinoma (*N* = 1), pancreatic carcinoma (*N* = 1), myelodysplastic syndrome (*N* = 1)	(63)	hairy cell leukemia, chronic myelogenous leukemias and non-hodgkins lymphomas	(64)
Alemtuzumab	Monoclonal antibody against CD52 on T and B cells; Depletion of peripheral lymphocytes via CDC and ADCC	**CARE MS I 5 year follow up:** RRMS (*N* = 376 year 1 and 2; *N* = 340–360 year 3–5): thyroid papillary carcinoma (*N* = 2), breast cancer (*N* = 1), keratoacanthoma (*N* = 1), non–small-cell lung cancer (*N* = 1), micropapillary thyroid carcinoma (*N* = 1) **CARE MS II 5 year follow up:** RRMS (*N* = 434 year 2 and *N* = 412 year 3): thyroid papillary carcinoma (*N* = 2), basal cell carcinoma (*N* = 1), melanoma (*N* = 1)	(65, 66)	T cell lymphomas, peripheral T cell lymphoma-not otherwise specified, T cell prolymphocytic leukemia, cutaneous T cell lymphoma and adult T cell lymphoma/leukemia; B cell malignancies: B cell Non-Hodgkin lymphoma and B cell chronic lymphotytic leukemia	(67)
Rituximab	Monoclonal antibody against CD20 on immature and mature B cells; depletion of CD20 positive B cells via CDC and ADCC	RRMS (*N* = 557), SPMS (*N* = 198), PPMS (*N* = 67): Basalioma (*N* = 2), Pyoderma gangrenosum (*N* = 1)	(68)	B cell lymphomas, Non-Hodgkin lymphoma, Burkitt lympoma, and B cell lymphoblastic leukemias	(69)
Ocrelizumab	Monoclonal antibody against CD20 on immature and mature B cells; depletion of CD20 positive B cells via CDC and ADCC	**OPERA 1 and Opera 2:** RRMS (*N* = 825): renal cancer (*N* = 1), melanoma (*N* = 1), and breast cancer (*N* = 2) in RRMS patients; **ORATORIO:** PPMS (*N* = 486) breast cancer (*N* = 4), basal cell cardinoma (*N* = 3), large cell lymphoma (*N* = 1), endometrial carcinoma (*N* = 1), metastatic pancreatic carcinoma (*N* = 1) malignant fibrous histyocytoma (*N* = 1)	(70, 71)	Relapsed/refractory follicular lymphoma	(72)
Dimethyl Fumerate	Modulates Nrf2 and glutathione levels in T cells; activates antioxidant genes	**DEFINE:** RMS (*N* = 826) basal cell carcinoma (*N* = 1), breast cancer (*N* = 1), cervical cancer (*N* = 1), transitional cell carcinoma (*N* = 1) **CONFIRM:** No neoplasms reported in BG-12 (DMF) treated group	(73, 74)	Lung adenocarcinoma, colon adenocarcinoma, melanoma	(75–77)

The role of natalizumab in cancer is currently being evaluated in clinical trials in pulmonary metastatic osteosarcoma (NCT03811886), and has previously been evaluated in multiple myeloma (NCT00675428) and acute-graft-vs-host disease (NCT02133924, NCT02176031).

#### Sphingosine 1-P Receptor (S1PR) Receptor Modulators: Fingolimod (Gilenya) and Siponimod (Mayzent)

S1PR modulators play an important role in both MS and cancer. Fingolimod (FTY720, Gilenya) was the first oral therapy approved for MS in 2010. Siponimod is the most recent FDA approved DMT for RRMS and secondary progressive MS (SPMS) as of April 2019. S1P modulator's mechanism of action has been extensively evaluated in preclinical studies in mouse models of MS as well as *in vitro* and *in vivo* models of tumorigenesis and transplant.

Fingolimod was originally synthesized from a natural compound myriocin, from a family of parasitic fungi, *Cordyceps sinclarii* (80), and shown to have potent immunosuppressant activity exceeding that of cyclosporine A (81, 82). Prior to this discovery, in traditional Chinese medicine, powder from another fungal subfamily, *Cordyceps sinensis*, had been widely used for its energy and “eternal youth” qualities (83). Fingolimod was subsequently demonstrated to be a prodrug analog of sphingosine, becoming phosphorylated into fingolimod-P by sphingosine kinase 2(S1K2), and interacting with S1P receptors on various cell types, outcompeting the native S1P. Although Fingolimod-P can bind with all S1PRs except S1P2, it has highest affinity for S1P4 (84). Following binding to S1PRs, fingolimod leads to internalization of the S1PR on T and B cells, preventing their egress from secondary lymphoid organs, such as mesenteric lymph nodes and Peyer's patches, and leading to peripheral lymphopenia (81). Similarly to fingolimod, siponimod binds to S1P1 receptors, leading to a decrease in peripheral immune cell egress from lymph nodes. Via its action on the S1P5 receptors in the CNS, siponimod is also thought to potentially contribute toward a decrease in extent and progression of neurodegeneration (85).

In competing with S1P, S1P modulators participate in an intricate cellular machinery of sphingolipids, major components of eukaryotic cell plasma membranes, which play an important role in cellular fate and cell signaling. Sphingolipids such as ceramide and sphingosine are important in apoptotic machinery of programmed cell death, while S1P is involved in cell proliferation, migration, angiogenesis, inflammatory responses, and lymphocyte trafficking. Dysregulation in sphingolipid metabolism allows cancer cells to escape cell death via increasing S1P signaling, altering expression of ceramide degrading enzymes, and upregulation of sphingosine kinases, such as sphingosine kinase 1 (SK1) (86). Hence, as a sphingosine analog, fingolimod has been studied in *in vitro* and *in vivo* for its potential anticancer effects. Indeed, fingolimod has been shown in preclinical studies to have anticancer activity in various cancer cell types, including bladder cancer (87), breast cancers (88–91), glioblastoma (92, 93), hepatocellular carcinoma (94–96), malignant mesothelioma (97), leukemia and lymphoma (98–104), lung cancer (105–107), liver cancer (108), pancreatic cancer (109), bladder cancer (87), renal cancer (110); glioma (111), gastrointestinal cancer (112), and ovarian cancer (113). Fingolimod has also been shown to be an important therapy sensitizer in several studies. For example, fingolimod demonstrates an additive effect with 5-fluorouracil, SN-38, and oxaliplatin (114), in colorectal cancer studies. It also leads to inhibition of tumor growth and induction of cancer cell apoptosis and mouse survival when used with cetuximab (115). Other anticancer effects of fingolimod include inhibition of metastasis in a mouse model of melanoma (116) and glioblastoma cell lines (93), and inhibition of microvessel formation in prostate tumor xenografts in mice (117). Fingolimod has strong immunosuppressive properties against Treg cells (118) that contribute to tolerance of malignant tumor cells (119) indicating fingolimod may have potential in post-transplant malignancies (120). Yet another potential anticancer mechanism of fingolimod is inhibition or degradation of SK1, which is upregulated in multiple cancers, including CNS (brain), gastrointestinal (colon, stomach, rectum, small intestine), genitourinary (ovary and uterus), pulmonary and breast (121). Overall, it is becoming clear that fingolimod has a multitude of anticancer effects in addition to its role in immunosuppression as an S1P modulator, that in part may be mediated via its unphosphorylated form (122).

**Figure 2:**
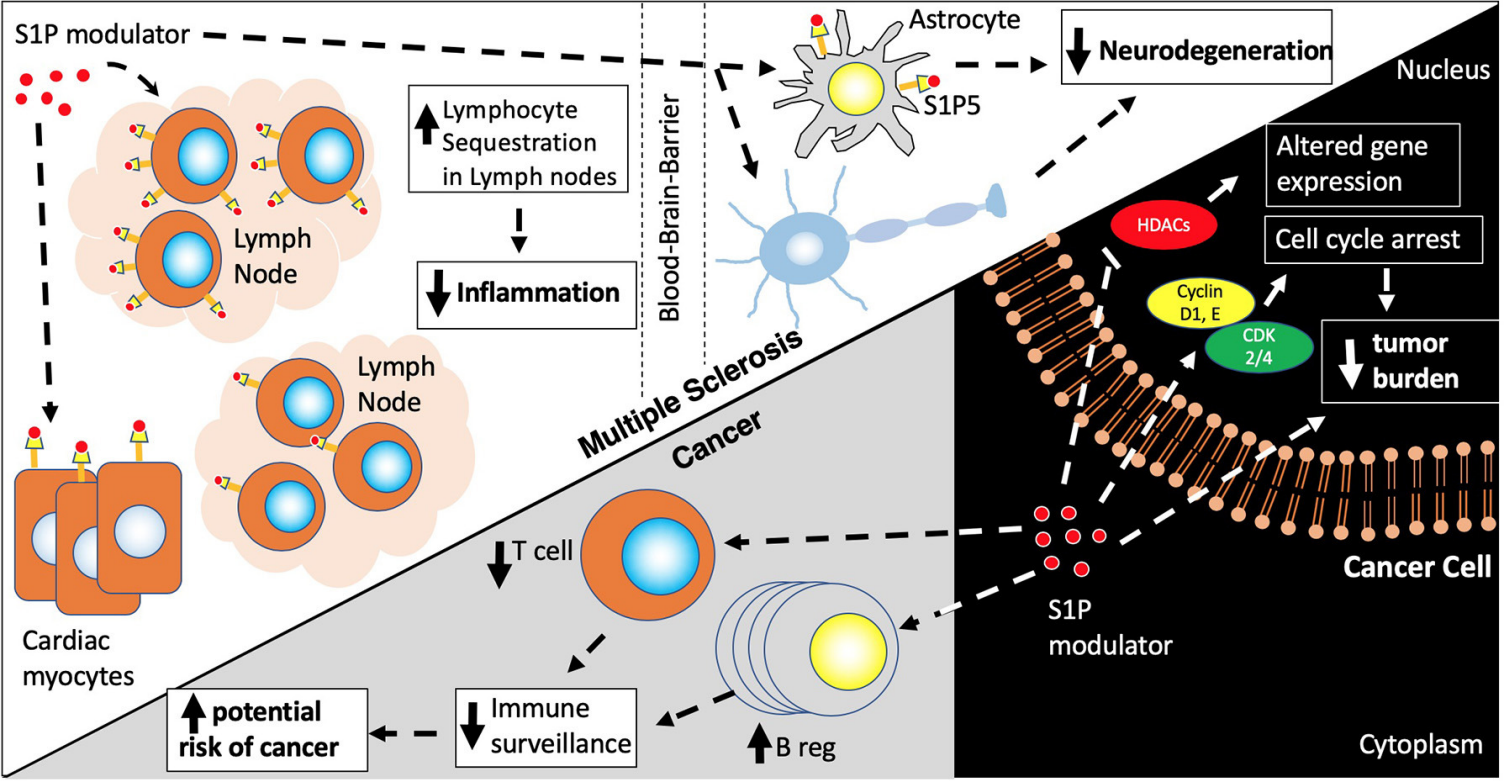
S1P modulators. S1P modulators, Fingolimod and Siponimod, decrease egress of naïve memory, Th17, CD4/CD8 T cells, and plasma B cells from lymph nodes leading to a decreased inflammatory response and decrease in neurodegeneration via actions on the S1P receptors in lymph nodes, astrocytes and oligodendrocytes. S1P modulators also have actions on other cell types including cardiac myocytes contributing to first dose bradycardia. In cancer cells, S1P modulators activate pathways involved in cell cycle arrest and cell death via actions on histone deacetylases (HDACs) and cyclin/CDK cell cycle proteins. However, due to their effects on Bregs (increased) and T regs (decreased) there is a risk of increasing cancer incidence due to decreased immune surveillance.

However, despite fingolimod's significant potential as an anticancer drug, there are several caveats that preclude its sole use as a cancer treatment. First, given fingolimod's effect on lymphocyte sequestration, decreased T cell surveillance may enhance potential cancer development (Figure 2). Additionally, upregulation of B regulatory cells (Bregs) and IL-10 could serve as another mechanism of fueling potential tumorigenesis (123). To this degree, there are a low number of fingolimod associated malignancies that have emerged in clinical trials (Table 1) and in the post-clinical trial era, including melanoma, basal cell carcinoma, breast cancer, squamous cell carcinoma, large B cell lymphoma, ocular lymphoma, Merkel cell carcinoma, cutaneous CD30+ T-cell lymphoma and multiple myeloma. Additionally, doses required for fingolimod's antitumor effects far exceed the dosing currently approved for fingolimod in MS and may lead to unwarranted side effects (124). Still, given its important role in tumor sensitization, Fingolimod could potentially be used as a sensitizer for cancer treatment alongside other cancer treatment strategies.

Perhaps in the future, other analogs of S1PR that are currently in different phases of preclinical and clinical trials, such as SKI-178 (125), vs. liposomal formulations for targeted delivery of fingolimod will maximize anti-cancer effects and minimize immunosuppressive and other side effects of fingolimod. In addition, fingolimod could be used as a sensitizer at a relevant dose in patients with MS who may be diagnosed with other cancers. These considerations may guide clinician decision not to discontinue therapy but to use it as an adjuvant to other cancer treatments.

A recent clinical trial has completed enrolling patients to evaluate fingolimod's role as a tumor sensitizer in patients with glioblastoma (NCT02490930).

## DNA Synthesis Inhibitors: Teriflunomide (Aubagio) and Cladribine (Mavenclad)

### Teriflunomide (Aubagio)

Teriflunomide was the second oral drug to be approved for MS in 2012. Teriflunomide is an active metabolite of lefluonomide and acts via reversible inhibition of the mitochondrial enzyme dihydroorotate dehydrogenase (DHODH) necessary for *de novo* synthesis of pyrimidines. By halting DNA and RNA synthesis, teriflunomide affects actively dividing cells, such as T and B cells. Teriflunomide was associated with rare cases of cervical carcinoma *in situ* and uterine leiomyosarcoma in clinical trials (60, 61) (Table 1). To date, there is one case report of possible association between lymphoma and terifluonomide (126). There are reports of possible other associations with breast and skin cancer in pharmacovigilance databases (127).

Both teriflunomide and leflunomide have been studied for their potent antitumor effects in different cancer types (62, 128) (Table 1). Interestingly, although DHODH is ubiquitously expressed, it is not overexpressed or mutated in malignant cells. However, its important role in cancer biology is thought to relate to malignant cells having a lower threshold for pyrimidine deprivation compared to non-malignant cells (129). Examples of anti-tumor mechanisms of teriflunomide and leflunomide include: down-regulation of anti-apoptotic proteins and growth factor receptors in cancer cells (62, 128), interruption of cancer cell survival signaling (130, 131), induction of cancer cell death (128, 132), abolishment of cancer stem cells (133), and cancer cell mitochondrial disruption (132). Teriflunomide may be an attractive anti-tumor medicine due to its efficacy at lower doses compared to other medications that inhibit DNA synthesis, such as methotrexate, and may thus avoid cumulative cytotoxic damage to the body (129). In particular, teriflunomide has been shown to improve basal cell carcinoma outcomes (134).

Leflunomide has been investigated in several clinical trials as an anti-cancer therapy, including metastatic triple negative cancers (NCT03709446), add-on therapy to mitoxantrone in stage IV prostate cancer (NCT00004071), add on to vemurafenib in metastatic melanoma (NCT01611675), as treatment for multiple melanoma (NCT02509052), anaplastic astrocytoma (NCT00003775), and glioblastoma multiforme (NCT00003293).

### Cladribine (Mavenclad)

Cladribine is one of the newest DMTs approved for MS. As of 2019, cladribine has been approved in Europe and most recently approved in by the FDA in the US April 2019 for treatment of RRMS and SPMS.

Cladribine was synthesized in the 1980's as an adenosine analog, with resistance to adenosine deaminase due to a substitution of a chloride at the 2′-hydrogen position. In its phosphorylated form inside cells, cladribine is unable to diffuse out of the cell membrane and thus becomes trapped intracellularly (135). Cladribine's incorporation into the DNA chain, results in chain termination and ultimate cell death. Interestingly, cladribine is cytotoxic not only to dividing cells, but also to resting cells, suggesting alternative mechanism of action in non-dividing cells such as caspase- dependent (136) and caspase-independent (137) mechanisms, via direct mitochondrial toxicity (137, 138), inhibition of DNA repair (139) and epigenetic alterations (140).

Cladribine initially was thought to be specific to lymphocytes, given the observation of isolated lymphopenia in patients with severe combined immunodeficiency syndrome (SCID). Later discoveries of cladribine's toxicity to monocytes and macrophages raised the question of potential of cladribine to treat myeloid malignancies. Initial studies on cladribine have been done in pediatric acute myeloid leukemia (AML) (141–143). Further studies in different cancers, including acute and chronic leukemias, mantle cell lymphoma, hairy cell leukemia, mucosa associated lymphoid tissue (MALT)-type lymphoma, and Langerhans cell histiocytosis have been done with combination therapies in both pediatric and adult populations (144–148). Cladribine's main adverse effects documented from the cancer studies, included significant immunosuppression associated with opportunistic infections such as herpes simplex, paresthesias, and possible secondary malignancy (149).

Cladribine was developed as a drug for MS given its lymphosuppressive actions, and in phase III trials in RRMS was shown to decrease disease activity in 45% of patients after 2 courses of treatment (63, 150). Due to a concern for inducing malignancy [a total of 10 cases in the active treatment arm of melanoma, ovarian carcinoma, pancreatic carcinoma, and myelodysplastic syndrome (63)] cladribine was rejected by FDA in 2010. However, a 2015 meta-analysis, comparing cancer risk in 11 phase III trials between different available DMTs at the time, including GA, natalizumab, dimethyl fumerate, tefiluonomide, and fingolimod, demonstrated that there was not a higher cancer risk in MS patients who were treated with cladribine, but rather that due to a placebo comparator, there was an exaggerated relative increased risk in the treatment groups (151). Other long-term studies in leukemia patients have also shown lack of an increase in secondary malignancies (147). Long-term monitoring for potential cancer side effects in MS patients on cladribine is warranted and has been recommended by EMA and FDA to determine the true cancer risk in this patient population.

## Monoclonal Antibodies: Alemtuzumab (Lemtrada) and B Cell Therapies: Rituximab (Rituxan, Mab Thera, Ritemvia) and Ocrelizumab (Ocrevus)

### Alemtuzumab (Lemtrada)

Alemtuzumab is a humanized monoclonal antibody directed against CD52, approved for MS in 2013. Treatment of alemtuzumab leads to significant depletion of T and B lymphocytes, natural killer (NK) cells, dendritic cells, granulocytes, and monocytes via several mechanisms (152). First, by activating C1q and generation of the membrane attack complex, alemtuzumab leads to complement-dependent cytotoxicity. Second, by activation of NK cells and macrophages through their IgG fragment C receptor, alemtuzumab contributes to antibody-dependent cellular cytotoxicity. Lastly, alemtuzumab can also result in induction of apoptosis. The final result of alemtuzumab is profound depletion of peripheral lymphocytes that occurs within hours-days post infusion and is sustained for up to 1 year (153). B cells tend to repopulate faster than T cells, contributing to an immune imbalance, which may explain some of the autoimmune side effects associated with Lemtrada (154).

As CD52 is expressed on the cell surface of both normal and malignant lymphocytes, alemtuzumab has been an important therapy for several cancer types for over 20 years prior to its approval as an MS therapy. In fact, alemtuzumab, under the name camcath, was used in the 1980's for the treatment of Hodgkin's lymphoma, and was FDA approved in 2001 for the treatment of chronic lymphocytic leukemia (CLL) (67). Over the years, alemtuzumab has been used to treat lymphomas and leukemias, including T cell lymphomas, peripheral T cell lymphoma-not otherwise specified (PTCL-NOS), T cell prolymphocytic leukemia (T-PLL), cutaneous T cell lymphoma (CTCL) and adult T cell lymphoma/leukemia (ATLL) as well as B cell malignancies, such as B-cell non-Hodgkin's lymphoma (B-NHL) and B-cell chronic lymphocytic lymphoma (B-CLL).

Several malignancies have been associated with alemtuzumab use in RRMS, including stage 1 thyroid papillary carcinoma with onset 10–41 months from the last infusion, basal cell carcinoma, breast cancer, melanoma, non-EBV-associated Burkitt' s lymphoma, and Castleman disease. Overall, out of 1,486 alemtuzumab treated-patients in 3 clinical trials, 29 patients developed malignancies, at variable times post treatment (Table 1).

### B Cell Therapies: Rituximab (Rituxan), Ocrelizumab (Ocrevus)

**Figure 3:**
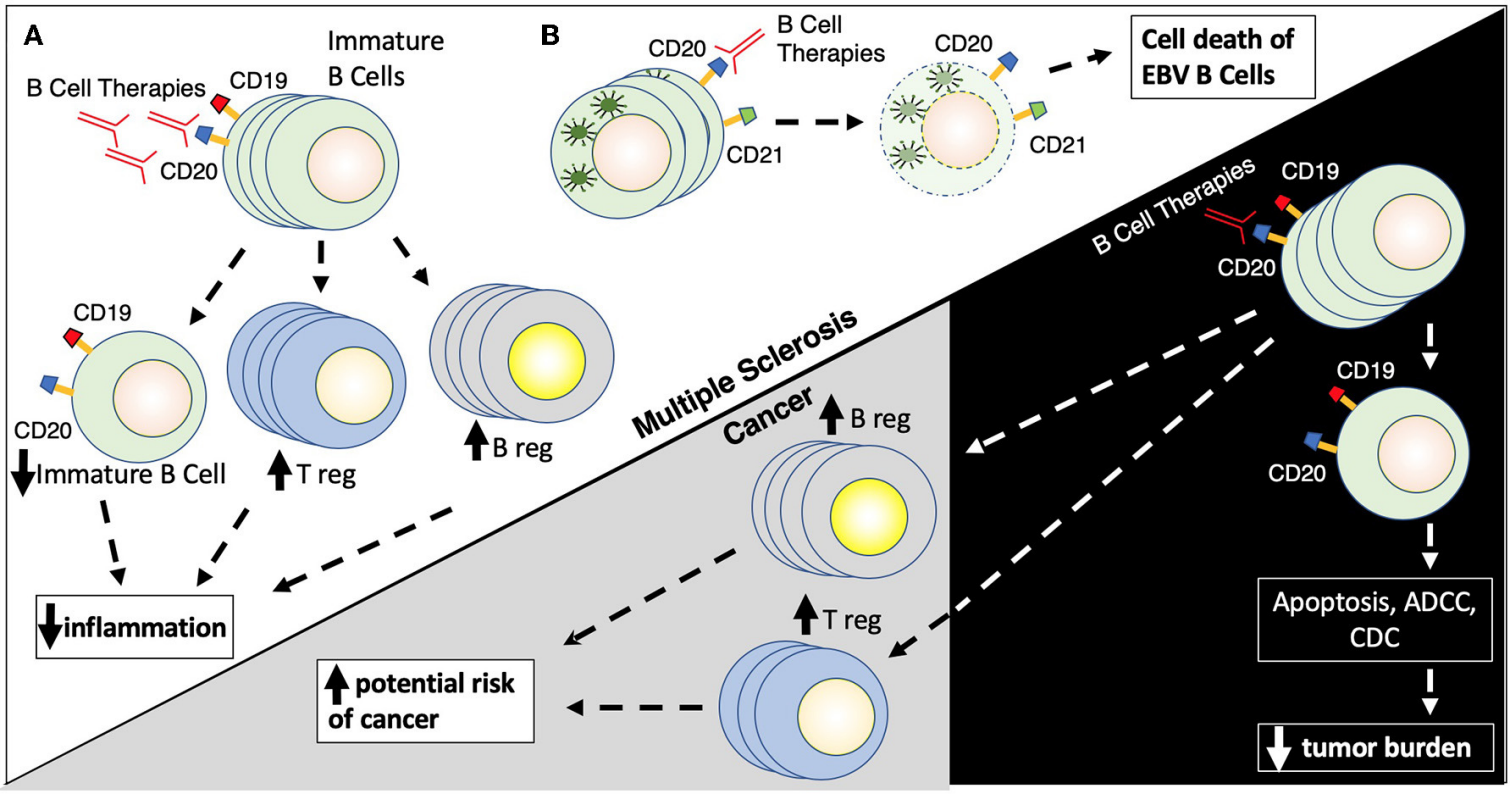
**(A,B)** B cell therapies. B cell therapies play a role in the treatment of both multiple sclerosis and cancer, but yet can also promote cancer development. Rituximab targets CD20 found on the surface of immature (naïve) B cells (CD19/CD20+) leading to their destruction by several mechanisms including apoptosis, antibody-dependent cell cytotoxicity, and complement directed cytotoxicity. Rituximab also promotes the proliferation of T regulatory cells (Treg) and B regulator cells (Breg) which results in reduced inflammation. Rituximab has similar actions in the context of cancer, where elimination of CD19/CD20+ B cells leads to a reduction of tumor burden in patients with B cell malignancies, while elevations in Breg and Treg populations can promote possible tumor formation.

Rituximab and ocrelizumab, anti-CD20 B cell depleting monoclonal antibodies, have become critical agents in the management of MS, underlying the importance of B cells in the pathophysiology of MS. Rituximab is a chimeric monoclonal antibody targeted against CD20, while ocrelizumab is a humanized anti-CD20 agent. Although not FDA approved in demyelinating diseases, rituximab is widely used in the treatment of MS and Neuromyelitis Optica. Ocrelizumab was FDA approved in 2017 for RRMS and primary progressive multiple sclerosis (PPMS) based on two large phase III trials (OPERA I and II) (71). Further anti-CD20 agents, currently in late stages of the development pipeline, include ofatumumab and ublituximab. The anti-CD20 therapies lead to elimination of CD20 expressing pre-B cells and mature lymphocytes via a variety of mechanisms, including antibody-dependent cellular cytotoxicity (ADCC), antibody-dependent cellular phagocytosis, complement-dependent cytotoxicity (CDC), cell death via apoptosis and decreased antibody production (155) (Figure 3).

B cells are well recognized for their role in modulating the immune response in cancer. Tumor metabolites, like leukotriene B4, can attract B cells to tumor sites and promote differentiation of B regs and T regs, and shifting the immune milieu from CD8 and T-helper 1 (Th1) to Th2-driven response (156). While a more regulatory immune environment is beneficial in autoimmune conditions such as MS, the shift to Breg/Tregs/Th2 prevents the host immune system from detecting and lysing tumor cells and could fuel cancer growth via IL35 and IL10 producing Bregs (Figure 3). In addition, another subset of B cells, CD5þ B cells, can promote several cancer cell types (157). Also, the presence of CD19+ B cells may be associated with worse outcomes in metastatic ovarian carcinoma (158).

Importantly, the CD20 cell surface marker is found on most malignant B cells. Thus, anti-CD20 therapies, such as rituximab have earned a prominent role in treatment of cancers, including B cell lymphomas, Hodgkin lymphoma, Burkitt lymphoma, and B cell lymphoblastic leukemias (69). Second and third generation anti-CD20 monoclonal antibodies, ocrelizumab and ofatumumab, have shown promise as alternative cancer therapies in patients with intolerance to rituximab (159). However, in contrast to rituximab, there is no long-term safety data on these newer second and third generation agents.

Despite the success of anti-CD20 monoclonal antibodies in cancer treatment, there are also several cancers associated with the use of anti-B cell therapies. For example, ocrelizumab was associated with several cases of malignancy in clinical trials, including cases of renal cancer, melanoma, and breast cancers. Overall, 4 malignancies were reported in OPERA 1 trial in RRMS patients and 11 malignancies were reported in PPMS patients in the ORATORIO trial (70).

Reasons for cancer predisposition on B cell therapies may have to do with the potentially protective role of B cells in tumor microenivronments as APCs and activators of NK and cytolytic T cells targeted toward lysis of tumors. A recent cancer profiling study has linked a B cell gene signature to fast proliferating tumors, such as breast cancers, suggesting that suppression of a B cell response in breast cancer could contribute to less favorable outcomes (160). Other studies have also demonstrated that B cell presence, and specifically CD20 B cells, are linked to a better prognosis in breast cancer (161, 162). Similarly, it has been demonstrated that co-presence of both CD20 B cells and CD8 T cells in the tumor environment improves survival in ovarian cancer (163). Interestingly, the timing of anti-B cell therapy may also dictate type of tumor response. For example, one study found that treatment of mice with anti-CD20 therapy prior to tumor challenge resulted in elimination of cancer metastasis. In comparison, treatment with anti-CD20 therapy post tumor challenge, enhanced tumor cell survival and metastasis (164). Thus, timing of anti-B cell therapy in context of cancer co-morbidities in MS may be an important factor in clinical decision making.

## Antioxidant Pathway

### Dimethyl Fumerate (Tecfidera)

**Figure 4:**
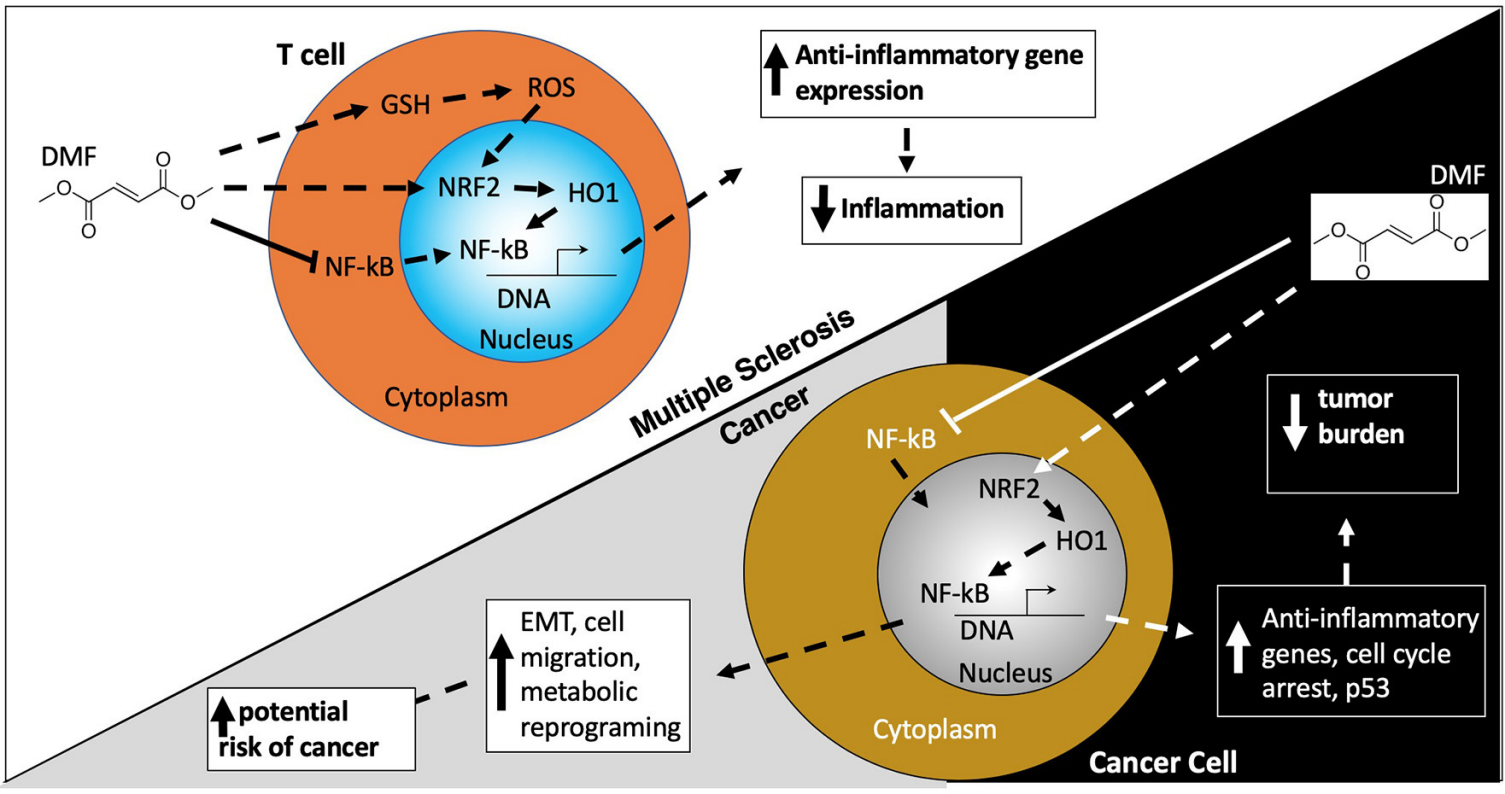
Dimethyl fumerate. Dimethyl Fumerate (DMF) ultimately enhances the activity of the transcription factor nuclear factor (erythroid-derived 2)-like 2 (NRF2) leading to transcription of anti-inflammatory genes, serving as the basis for DMF's beneficial effects in MS patients. In cancer cells, these same actions together with alterations in cell cycle protein and p53 expression, serve to attenuate cancer growth and proliferation. However, paradoxically DMF can also promote epithelial to mesenchymal transition (EMT) which enhances migration and metastasis. DMF may also alter the metabolic environment in cancer cells which aids their survival, growth, and proliferation.

Initially used in the treatment of psoriasis, dimethyl fumerate (DMF) was approved for use in MS in 2013. Although the mechanism of action of DMF is not completely understood in MS, DMF is thought to be an immunomodulatory therapy that decreases inflammatory T cells via increasing nuclear factor erythroid 2–related factor 2 (Nrf2) and glutathione (GSH), the cell's regulators of anti-oxidant response (Figure 4) (165). DMF is derived from fumaric acid and is metabolized into monomethyl fumerate (MMF), the active component responsible for DMF's anti-inflammatory and antioxidant effects. DMF was associated with a low level of malignancies in the DEFINE and CONFIRM clinical trials, accounting for <1% of all adverse events (Table 1).

A number of preclinical studies have explored the benefits of DMF and MMF in anti-cancer activity via impact on the cell's apoptotic machinery. DMF has been shown to be cytotoxic in patient and mouse derived cancer cell lines from lung and colon adenocarcinoma with KRAS mutations, likely via effect on decreasing activity of the Nrf2/DJ-1 antioxidant pathway and contributing to cancer cell death (75, 76). Several studies have illustrated positive role of DMF in melanoma tumor suppression via mechanisms interfering with cancer cell proliferation or contributing to apoptosis and cell cycle inhibition. For example, DMF was shown to decrease melanoma metastasis as well as lymph node metastasis in a SCID mouse model (166, 167). In another study, DMF inhibited growth and metastasis of melanoma tumors by suppressing metalloproteases and inhibiting entry of nuclear factor B and its transcription factor p65 into the nucleus (168). Study of DMF's role in cell cycle arrest, demonstrated that DMF inhibited melanoma cell line proliferation via interference with cell cycle proteins and upregulation of tumor suppressor, p53 (169). In a separate study, DMF induced cell death in colon carcinoma cells, via depletion of GSH and activation of mitogen-activated protein kinase (MAPK) (170). In a breast cancer cell line, it was found that via blockade of transcription factor p65 in the nuclear factor B (NFK B) pathway, DMF is able to retard tumor proliferation and xenograft tumor growth (171). Both DMF and MMF were found to retard tumor growth in primary human glioblastoma cell lines alone or in combination with other anti-tumor medications in the proteasome inhibitor family (e.g., velcade and carfilzomib) (77).

As a result of these pre-clinical studies, several clinical trials are currently underway to evaluate the role of DMF in cancer, including a trial in refractory leukemia/lymphoma, NCT02784834; glioblastoma, NCT02337426; and Cutaneous T Cell Lymphoma, NCT02546440.

## Conclusions and Future Directions

The salient role of the immune system is to defend the body from invasive pathogens and cancers, while preserving tolerance to self-antigens. Both cancer and autoimmune disorders, such as MS, result from immune dysregulation. In MS, the immune system reacts too strongly in self-protection, resulting in demyelination of the central nervous system. Cancer cells adapt to either evade standard immune checks and balances or directly influence an immune phenotype conducive toward malignant proliferation, such as via recruitment and differentiation of Tregs, B regs, CD5þ B, and CD19+ B cells within the tumor microenvironment.

An important goal in successful management of MS patients is to resolve how to (i) best therapeutically manipulate the immune system without promoting cancer and how to (ii) treat MS patients who develop cancer while on a DMT. It is noteworthy that many of the current DMTs have potential anti-tumor mechanisms. Examples, such as basal cell carcinoma improvement with terifluonomide treatment (134) suggest that a DMT should not be stopped at cancer diagnosis, but rather each individual's case should be considered in context of their specific risk factors for cancer. Thus, in making treatment decisions for MS patients with cancer, it is imperative that clinicians consider the potential mechanism of action of available DMTs to determine how best to manage both the cancer risk and the risk of rebound MS.

In addition, it is also critical that clinicians think about patient-specific risks. Both pre-clinical and clinical studies demonstrate that not all individuals with MS develop cancer and cancer side effect of MS DMTs are tissue specific (Table 1). What may account for this patient heterogeneity is not well-understood, but likely has to do with context dependent cues, such as an individual's cultural background and genetics, being male vs. female, diet, composition of the gut microbiome, length of treatment on DMTs, and likely other not yet recognized factors.

Toward the idea of personalized risk assessment, it is likely important that sex differences should be considered in treatment of MS and cancer. An important example is recent data on immune checkpoint inhibitors, demonstrating that there is a significant difference in immune responses in men and women, with the higher possibility for males to benefit from cancer immunotherapies (172). Some MS studies have also hinted at sex-specific responses to DMTs, potentially due to sexually dimorphic immune responses (173, 174). While more studies are necessary to determine exact cancer risks that male and female MS patients may experience on specific DMTs, it is prudent that sex differences in response to DMTs should be considered at initiation or discontinuation of DMTs when a patient develops cancer.

Another potential factor that may differentially determine a person's predisposition for and treatment of MS (175–177) and cancer (178–180) is the gut microbiome. The human gut microbiome consists of bacteria, fungi, archaea, protozoa, and viruses that have evolved symbiotic relationship with the human host (181). Trillions of gut microbiota mediate multiple functions for the host, and in particular help shape the immune system (182–184). In MS, studies have shown that transfer of fecal microbiota transplant (FMT) from patients with MS to recipient mice can induce experimental autoimmune encephalomyelitis (EAE), MS-like disease in mice (185–187), suggesting that gut microbes are a vital aspect of autoimmune induction. Likewise, cancer phenotypes can be transferred via FMT (188). Gut bacteria can also help to fight autoimmunity and cancer by modulating host immunity (189) and improving outcomes of immunotherapy treatments (190). Interestingly, butyrate producing bacteria have been shown to be beneficial in both MS (191, 192) and cancer (193, 194), via effects on cellular proliferation and apoptosis as well as immune effects on the blood brain barrier and remyelination. Given the importance of the gut microbiome in both autoimmunity and cancer, it is exciting to view the manipulation of the gut microbial populations as an avenue for future management of MS and cancer. There are still many questions about how gut bacteria can influence the course of immunity at organs distant from the gut, and whether same bacteria may influence immunity differentially in autoimmune conditions vs. cancer. For example, *Bifidobacteria, Akkermansia*, and *Bacteroides* are increased in patients with MS, but may lead to anti-tumor outcomes in cancer patients (195, 196). Research to better delineate which bacterial species are most conducive to promoting favorable vs. unfavorable immune outcomes in MS and cancer, will help to pave the road toward more precise risk assessment and manipulation of the gut microbiome in patients with MS and cancer.

In summary, given the wide array of currently available DMTs with potential to promote and treat cancer, it is important for clinicians to be aware of the risks and benefits of prescribing these agents to MS patients, particularly to patients with a history of cancer. Future efforts should be directed toward developing a clearer understanding of the relative risk and incidence of cancer in patients taking specific DMTs. One possible solution would be creation of a curated data base and establishment of uniform standards and guidelines on reporting and management of patients with MS and a history of cancer who are candidates for DMTs. In addition, there is a need for development of clinical recommendations for frequency and type of monitoring for malignancy screening on individual DMTs as well as research and recommendations for how to transition MS patients to alternate DMTs if cancer arises. Further research on sex differences and the role of the environmental factors, such as the gut microbiome in MS and cancer, will allow for an integrated pathway toward treating both conditions in a personalized way.

## Author Contributions

EM and ML: take responsibility for the integrity of the data and the accuracy of the data analysis, acquisition and interpretation, and the critical revision of the manuscript for important intellectual content. EM: concept and design and drafting of the manuscript.

### Conflict of Interest

EM has served as a consultant for EMD Serono and Genentech. The remaining author declares that the research was conducted in the absence of any commercial or financial relationships that could be construed as a potential conflict of interest.
